# A candidate-variant association study of self-reported asthma in mothers of the Cebu Longitudinal Health and Nutrition Survey (CLHNS) cohort

**DOI:** 10.1186/s12863-026-01419-5

**Published:** 2026-06-18

**Authors:** Alvin E. Lirio, Jing Chen, Paulita Duazo, Nanette Lee, Linda Adair, Martin D. Tobin, Catherine John, Anna L. Guyatt

**Affiliations:** 1 Division of Public Health and Epidemiology, University of Leicester, Leicester, UK; 2 UNC Chapel Hill Genetics Department, Chapel Hill, USA; 3 USC Office of Population Studies, University of San Carlos, Cebu City, Philippines; 4 University Hospitals of Leicester NHS Trust, Leicester, UK

**Keywords:** Asthma, Filipino, Candidate-variant association study, Genetic risk scores

## Abstract

**Background:**

Asthma is an obstructive respiratory disease with greatest morbidity in low- and middle-income countries: 15% of the Filipino population are estimated to have asthma, half of whom have inadequately controlled disease. There are many known genetic associations with asthma, but lack of representation across ancestries means that understudied populations do not yet stand to benefit equally from genomic research. We performed the first genetic association study of asthma in a Filipino population. We performed a candidate-variant association study in 1,678 unrelated mothers (129 with self-reported asthma) of the Cebu Longitudinal Health and Nutrition Survey (CLHNS). Using the largest published multi-ancestry asthma GWAS (Global Biobank Meta-Analysis Initiative, GBMI), we selected sentinel single-nucleotide polymorphisms (SNPs) associated in the multi-ancestry analysis (*P* < 5 × 10^− 8^), and in GBMI East Asian (EAS) participants (*P* < 5 × 10^− 6^). SNPs (or proxies r^2^ > 0.8) were tested for association with asthma, adjusting for age and 15 principal components. Variants were then aggregated into a genetic risk score (GRS), weighted by effect sizes reported in the EAS GBMI asthma GWAS. We examined whether the identified top signals from GBMI EAS GWAS were associated in our study.

**Results:**

Twenty-seven SNPs were analysed. Only one intronic variant in *SMAD3* was associated with asthma at a Bonferroni-corrected threshold (rs17293632, OR 1.80, 95%CI 1.28–2.54, *P* = 0.0008). *SMAD3* is involved in TGF-β signalling, related to airway remodelling in asthma. The GRS was associated with increased odds of asthma (OR per weighted allele increase 1.07 [95%CI 1.01–1.14], *P* = 0.022). Five of the 37 top signals in the EAS GWAS were associated in our study, although none of these five signals reached a Bonferroni *p*-value threshold of *P* = 1.35 × 10^− 3^.

**Conclusions:**

Despite the small sample size, we detected a known SNP-asthma association in CLHNS using a candidate-variant approach, and demonstrated the value of aggregating variants into GRS when power is limited (using confidently ascribed weights from large GWAS). However, we were underpowered to undertake a GWAS, and thus to discover novel associations. Commitment by the genomics field as a whole to cohort development and investment in understudied populations will be key to addressing inequity in asthma genetic research.

**Clinical trial number:**

Not applicable.

**Supplementary Information:**

The online version contains supplementary material available at 10.1186/s12863-026-01419-5.

## Background

Asthma is an obstructive respiratory disease characterised by reversible airflow obstruction due to airway narrowing (bronchoconstriction), airway wall thickening, and increased mucous secretion [1, 2]. Asthma morbidity is greatest in low- and middle-income countries: 15% of the Filipino population are estimated to have asthma, half of whom have inadequately controlled disease [3].

As well as proposed environmental factors that may cause or exacerbate asthma, such as ambient air pollution, respiratory infections, smoking, and allergies, asthma has a substantial genetic component [1, 2]. Using genome-wide association studies (GWAS), SNP heritability estimates for childhood- and adult-onset asthma stand at 25.6% and 10.6% [4], and over 200 risk variants have now been identified for asthma from GWAS [5].

Although recent advances have been made to improve representation of diverse populations in asthma genetic research, the vast majority of GWAS have been conducted in participants of European ancestry [6], with no studies to date in a Filipino population. A lack of ancestry representation means that understudied populations do not yet stand to benefit equally from genomic research [7]. Therefore, we aimed to conduct the first genetic association study of asthma in mothers from the Cebu Longitudinal Health and Nutrition Survey (CLHNS), a Filipino population-based cohort.

## Methods

The CLHNS cohort was established in 1983 and recruited women (and their offspring) living in Metropolitan Cebu, Philippines. CLHNS comprises a broad collection of information, including demographic and socio-economic data, genomic data, and self-reported health conditions, including respiratory disease [8].

DNA was extracted from fasting blood samples collected from CLHNS mothers in 2005. Full details of genotyping, imputation, and quality control are provided in the Supplementary Note, and have been described previously [9]. However, in brief, mothers were genotyped using the Affymetrix Genome-Wide Human SNP Array 5.0 at Vanderbilt University Medical Centre, Nashville, TN, USA [9]. Genotype data were available for 1,799 CLHNS mothers at 413,220 SNPs (384,311 after quality control). Imputation was undertaken using the 1000Genomes (phase 3.v5) reference panel, with 1000G East Asian (EAS) ancestry participants as the reference population. Second-degree relatives or closer were excluded from analysis, using a kinship threshold of > 0.0884 [10].

We studied 1,678 unrelated mothers and imputed genetic data passing quality control (129 with self-reported asthma, 1,549 controls - see Supplementary Fig. 1). Cases were those recorded with an affirmative answer to the question: “Did mother have asthma since 2002/2005 (or last visit)?” (assessed via interviews with mothers by research professionals at the 2007 CLHNS follow-up). Although data on respiratory conditions were collected since 1994, these offered non-specific details and were collectively grouped into single categories (e.g., mothers’ health condition and children’s respiratory illness) until 2007, when self-reported asthma for mothers was specifically investigated.

Since the small sample size available precluded a genome-wide association study (see Supplementary Table 1), we performed a candidate variant association study, using the largest multi-ancestry GWAS of asthma published to-date (Global Biobank Meta-analysis Initiative, GBMI, (153,763 cases and 1,647,022 controls) [11]. Independent genome-wide significant signals SNPs from this analysis (*P*<5 × 10^− 8^) were subset to those that were also associated with asthma in the EAS subgroup (18,549 cases and 322,655 controls) at *P*<5 × 10^− 6^, *and* that were also available in CLHNS (either directly, or using a proxy at r^2^ > 0.8). Proxies were identified using LDLINK R software [12]. We used PLINK2.0 to check that that all SNPs were independent in CLHNS at r^2^ < 0.1.

Single variants were tested for association with asthma in CLHNS, using logistic regression, specifying an additive genetic model, and adjusting for age, and 15 principal components of genetic ancestry. PLINK2.0 (https://www.cog-genomics.org/plink/2.0/) was used to run the analysis. A Bonferroni-corrected *p*-value threshold was applied (0.05/N SNPs tested). Nearest genes to analysed SNPs were annotated with ANNOVAR (https://annovar.openbioinformatics.org/en/latest/).

We assessed whether a weighted genetic risk score of the variants studied was associated with asthma, using the same covariates as for single-variant testing. Weights were taken from the GBMI EAS asthma GWAS [11], and transformed so that a one unit increase in GRS corresponded to one weighted allele, i.e. the minimum possible score was 0 and the maximum was twice the number of SNPs studied. Genetic risk scores were calculated using the --score flag of PLINK2.0, and regression analysis was run using R version 4.1.0 software.

Finally, we examined whether the identified top signals from GBMI EAS GWAS [11] were associated in our study. All SNPs in the GBMI EAS GWAS meeting the genome-wide significance threshold *P*<5 × 10^− 8^ entered sentinel selection, using a distance-based approach (± 500 kb of the variant with the lowest *p*-value [11]). Then, we performed a look-up of the top signals from the EAS GWAS by comparing *p*-values and effect sizes for the sentinel variants in our study.

## Results

Of the 179 independent sentinels (*P*<5 × 10^− 8^) associated with asthma in the GBMI multi-ancestry GWAS [11], 162 were analysed in the GBMI EAS subset of participants. Among these 162 variants, 74 variants were directly available in the CLHNS cohort, 85 had appropriate proxy variants and 3 had no available proxies. Of the 159 with either directly available or with suitable proxy in CLHNS, 27 were associated with asthma at *P*<5 × 10^− 6^ in the EAS subgroup of the GBMI asthma GWAS (10 variants directly available and 17 suitable proxy in CLHNS – see Supplementary Table 2).

Of the 27 variants studied, only rs17293632-T showed association with self-reported asthma (OR 1.80, 95%CI 1.28–2.54, *P* = 0.0008) at a Bonferroni-corrected threshold of *P* < 0.002 (i.e., a Bonferroni corrected threshold of 0.05/27) (see Table 1). SMAD Family Member 3 (*SMAD3*) is the nearest gene to the sentinel variant. This gene is expressed in various cells and tissues including thyroid, muscle, blood, CD 4 + T-cell, lung, and others [13], and is associated with the transforming growth factor-beta signalling pathway, and regulation of cell proliferation [14]. 15 out of the 27 variants tested in a candidate variant association analysis, showed a consistent effect direction with those reported in GBMI EAS GWAS, which included rs17293632-T near *SMAD3* (see Table 1).

**Table 1:** Comparing estimates and direction of effects of the 27 variants tested in a candidate variant association analysis of self-reported asthma in mothers of the CLHNS cohort with the GBMI EAS subgroup

rsID	Nearest Gene (variant type)	Chr: Pos: EffectAllele: OtherAllele	Effect allele frequency	CLHNS			East Asian GWAS			Effect Direction
				OR	95% CI	*P*-value	OR	95% CI	*P*-value	
rs1289273	*ITPKB (intronic)*	1:226909233:A: G	0.162	0.85	0.59–1.22	0.388	1.07	1.05–1.10	1.059e-07	-
rs13416555	*LINC00299 (intronic)*	2:8441735:C: G	0.229	0.73	0.53–1.02	0.064	0.92	0.89–0.94	1.336e-11	+
rs17046490	*NCOA1 (intronic)*	2:24945954:A: G	0.099	1.54	1.06–2.23	0.023	0.93	0.90–0.96	2.132e-06	-
rs72823632	*IL1RL1 (intronic)*	2:102931534:C: T	0.014	0.28	0.04–2.05	0.210	0.93	0.90–0.96	2.719e-06	+
rs4675360	*CD28 (intronic)*	2:204584456:A: T	0.277	1.11	0.83–1.47	0.482	0.92	0.90–0.94	4.845e-13	-
rs35305862	*D2HGDH (intronic)*	2:242700770:A: G	0.007	1.67	0.49–5.74	0.416	0.86	0.83–0.89	2.938e-17	-
rs35570272	*GLB1 (intronic)*	3:33047662:G: T	0.331	0.90	0.69–1.20	0.481	1.07	1.05–1.09	5.857e-09	-
rs4505848	*KIAA1109 (intronic)*	4:123132492:A: G	0.219	1.13	0.85–1.51	0.402	1.07	1.05–1.09	4.834e-09	+
rs2289276	*TSLP (intronic)*	5:110407507:C: T	0.243	1.07	0.80–1.44	0.630	1.14	1.11–1.16	2.400e-28	+
rs20541	*IL13 (exonic)*	5:131995964:A: G	0.394	0.92	0.70–1.20	0.530	0.93	0.91–0.95	4.568e-10	+
rs10068717	*NDFIP1 (intronic)*	5:141494934:C: T	0.492	0.93	0.71–1.20	0.574	1.06	1.04–1.08	8.142e-07	-
rs9270911	*HLA-DRB1 (intergenic)*	6:32572202:C: T	0.202	1.14	0.84–1.55	0.401	1.10	1.08–1.13	2.977e-18	+
rs10266726	*LOC105375130 (intronic)*	7:3146081:C: T	0.378	0.99	0.76–1.29	0.935	1.07	1.04–1.09	1.969e-07	-
rs7780222	*ABCB5 (intergenic)*	7:20557179:A: G	0.391	1.11	0.85–1.44	0.445	0.93	0.91–0.96	1.671e-08	-
rs16902848	*LINC00824 (intergenic)*	8:129399732:C: T	0.165	0.87	0.61–1.26	0.468	0.94	0.92–0.96	1.300e-06	+
rs7040995	*GADD45G (downstream gene)*	9:92226172:C: G	0.356	0.85	0.64–1.11	0.236	1.06	1.04–1.09	5.670e-07	-
rs2589559	*GATA3 (intergenic)*	10:9061370:C: T	0.109	1.24	0.84–1.81	0.274	0.86	0.83–0.88	6.512e-26	-
rs10995249	*ZNF365 (intronic)*	10:64396916:C: T	0.434	0.85	0.66–1.10	0.226	0.94	0.92–0.96	9.834e-09	+
rs7126418	*LRRC32 (intergenic)*	11:76292573:A: T	0.311	1.03	0.78–1.36	0.847	1.07	1.04–1.09	2.125e-08	+
rs11639084	*RORA (intronic)*	15:61066516:C: T	0.235	0.75	0.55–1.04	0.083	0.89	0.86–0.91	1.685e-16	+
*rs17293632*	***SMAD3 (intronic)***	***15:67442596:C: T***	***0.116***	***1.80***	***1.28–2.54***	***0.0008***	***1.15***	***1.08–1.21***	***3.559e-06***	***+***
rs3024548	*IL4R (intronic)*	16:27354531:C: G	0.421	0.96	0.74–1.26	0.775	1.10	1.08–1.13	8.924e-18	-
rs3744253	*NAA38 (intronic)*	17:7786699:G: T	0.110	0.74	0.47–1.18	0.207	1.08	1.05–1.12	1.165e-08	-
rs883770	*GSDMB (intronic)*	17:38063381:C: T	0.244	0.88	0.65–1.19	0.401	0.89	0.87–0.91	1.634e-20	+
rs2671654	*LOC102724596 (intergenic)*	17:47468011:A: G	0.342	0.87	0.66–1.15	0.340	0.93	0.90–0.95	1.302e-07	+
rs57631119	*SMAD2 (intergenic)*	18:45473449:C: T	0.222	1.24	0.93–1.67	0.147	1.07	1.04–1.10	5.292e-07	+
rs10416530	*MUC16 (intergenic)*	19:9129660:C: T	0.472	0.91	0.70–1.18	0.472	0.92	0.90–0.95	1.372e-09	+

Chr – chromosome, Pos – base-pair position, GRCh37 – Genome Reference Consortium Human Build 37; OR– odds ratio; 95% CI – confidence interval;

The threshold for statistical significance was *P* = 1.85 × 10^− 3^, calculated as 0.05 divided by 27, the number of SNPs tested

Effect direction: Symbols denote direction of effect estimates from CLHNS and EAS GWAS presented in the table, in the same order. ORs are aligned at the same effect allele (‘+’ means consistent direction of effect, ‘-’ means opposite direction of effect)

Nearest genes were annotated using online databases including ANNOVAR (https://annovar.openbioinformatics.org/en/latest/), NCBI (https://www.ncbi.nlm.nih.gov/snp/), and Open targets genetics (https://genetics.opentargets.org/Variant/)

When testing for the association between the weighted GRS of 27 SNPs and the asthma outcome (Supplementary Table 3), we observed an odds ratio for self-reported asthma of 1.07 (95%CI 1.01–1.14, *P* = 0.022), per weighted allele increase in the GRS.

Five of the 37 SNPs reported as associated with asthma in the GBMI EAS GWAS [11] were associated at *P* < 0.05 in this candidate variant association analysis of self-reported asthma in CLHNS cohort. Although none of these five signals reached a Bonferroni corrected p-value threshold of *P* = 1.35 × 10^− 3^, rs35570272-G (near *GLB1*, intronic), rs11639084-G (near *RORA*, intronic), and rs3744253-T (near *NAA38*, intronic) showed consistent direction of effects, while rs2289276-T (near *TSLP*, intronic) and rs7126418-C (near *LRRC32*, intergenic), showed opposite effect directions. (See Supplementary Table 4).

## Discussion

Despite the small number of asthma cases in the CLHNS cohort, we were able to detect a known asthma genetic variant using a candidate-variant approach, and demonstrated the value of aggregating variants into risk scores (using weights from a very large asthma GWAS [11], which should be precisely estimated) when power is limited. Our study represents the first genetic association study of respiratory disease in a Filipino population.

*SMAD3* was one of the first signals identified in GWAS of asthma [15], with a large effect size, making it easier to identify in a small sample. It is important in the transforming growth factor beta (TGF-β) signalling pathway related to airway remodelling (e.g. subepithelial fibrosis) in asthma [14], alongside other genes, regulating the proliferation of airway smooth muscle cells and bronchoconstriction [16]. Whilst we were able to demonstrate an association between a variant in *SMAD3* (rs17293632, intronic, MAF 12%) in the CLHNS cohort, the lack of association at other variants in single-variant testing is likely to reflect poor statistical power, especially since we found an association when variants were aggregated into a risk score. Despite using a self-reported outcome, these results suggest that the phenotype studied in CLHNS is likely to be capturing a similar phenotype to the asthma phenotypes included in the GBMI analysis, and that the genetic architecture of asthma in the participants studied may be similar to that of the GBMI EAS subgroup.

We were underpowered to undertake a *genome-wide* study, and thus to discover novel asthma variants in a Filipino population. This highlights the well-documented global equity issue of the genetic epidemiology field, since without support and resources to develop genetic studies in understudied populations, discovery of novel variants via GWAS, may allow for better risk prediction in diverse populations, which may not be possible due to power constraints. Incorporating a larger number of genetic variants and accounting for diverse ancestries would be a valuable next step. In particular, methods such as PRS-CS and LDpred [17], especially in their multi-ancestry or cross-ancestry implementations, have shown improved performance across populations and could help to develop polygenic scores that are more transferable and relevant to non-European and admixed groups [17].

## Conclusions

Commitment to cohort development and investment in understudied populations by the genomics field as a whole will be key to addressing inequity in asthma genetic research. Ultimately, improving the understanding of the genetic determinants of asthma in understudied populations may help inform accurate risk prediction in these populations and make the applications of genomic research more equitable, as well as contributing to overall understanding the biological pathways, which may inform drug development, eventually enhancing preventive and therapeutic measures across diverse populations.

## Supplementary Information

Below is the link to the electronic supplementary material.

Supplementary Material 1

## Data Availability

CLHNS data is managed by the Carolina Population Center, University of North Carolina at Chapel Hill. The CLHNS ‘dataverse’ is an open-source data file storage for the CLHNS cohort comprising data from every time-points from 1983 until 2009 for mothers, offspring, and households. This publicly available resource (https://dataverse.unc.edu/dataverse/cebu) provides a broad collection of information including demographics, socio-economics, and health conditions, except for restricted data (including genetic data), which were obtained upon the approval of a proposal submitted to the CLHNS data access committee, who also approved the analyses undertaken, and facilitated secure linkage of datasets as required.
