# Supplementary material for: A candidate-variant association study of self-reported asthma in mothers of the Cebu Longitudinal Health and Nutrition Survey (CLHNS) cohort
Source: BMC Genom Data. 2026 Jun 18;27:49. doi: 10.1186/s12863-026-01419-5 (PMC13308225; doi:10.1186/s12863-026-01419-5)
Supplement: Supplementary file 1 — Supplementary Material 1 [file 12863_2026_1419_MOESM1_ESM.docx]

# Supplementary Information

## Supplementary Note: Genotyping, imputation and associated quality control

DNA was extracted from fasting blood samples taken from CLHNS mothers in 2005. The mothers were genotyped using the Affymetrix Genome-Wide Human SNP Array 5.0 performed at the Vanderbilt Microarray Shared Resource at Vanderbilt University Medical Centre, Nashville, TN, USA^1^. The genotyped data for 1,799 CLHNS mothers’ samples and 413,220 SNPs were received from the University of North Carolina-Chapel Hill CLHNS investigators. Before receipt, samples that were highly related to multiple other participants were removed, as were samples with a call rate of <97%, and duplicate samples. SNP-wise quality control included the following criteria: exclude if (i) Hardy-Weinberg Equilibrium (HWE) P<10-8, (ii) minor allele frequency (MAF)<0.01, and (iii) SNP-wise call rate ≤90%. Once combined with phenotype data, there were 1,721 with non-missing genotype, age, and asthma status.

Using PLINK, the strand orientation, position of variants, alleles (reference and alternate) assignment, and frequency differences (i.e., the cohort allele frequency compared to the reference panel allele frequency) were examined. SNPs were excluded when: (i) they were palindromic (A-T or G-C) with a MAF >0.4, (ii) SNPs had differing alleles, (iii) SNPs had a difference in allele frequency that was greater than >0.2 when compared with the 1000Genomes reference panel, and (iv) SNPs were not in the 1000Genomes reference panel. After quality control, 384,311 genetic variants remained. Imputation was undertaken using the 1000Genomes (phase 3.v5) reference panel, with 1000G participants designated as being of East Asian (EAS) ancestry specified as the reference population. (Supplementary Figure 1).

A subset of quality-controlled SNPs (autosomal, non-palindromic SNPs, excluding the HLA regions; all MAF ≥1%, HWE P≥10-6 or ≤5% missing SNPs) in low linkage disequilibrium in the CLHNS sample was selected and combined with genotype data from the 1000G population data (as the reference panel). Cryptic relatedness was assessed using Kinship-based INference for Genome-wide association studies (KING) software (a kinship threshold of >0.0884 was used to exclude individuals who were second-degree relatives or closer)^2^. Then, principal components were constructed to capture population substructure among CLHNS mothers using the parameters within smartpca program from the EIGENSOFT package (https://data.broadinstitute.org/alkesgroup/EIG6.1/), specifying to utilise only the reference samples’ SNP loadings.

## Supplementary Figure 1 Data preparation for CLHNS sample used for genetic analysis

| 28,000 household surveyed from 33 barangays (17 urban, 16 rural area)  3,327 pregnant women interviewed for baseline information  Excluded:   - 26 multiple births - 42 still births/miscarriages - 17 drop outs - 136 out migrants - Lost to follow-up   3,080 single births from May 1983 to April 1984 were followed up  1,962 CLHNS mothers had data on age and self-reported asthma   - Age was derived from 2002 clinic visit records - Self-reported asthma data were from the 2007 clinic visit records   - 147 mothers with self-reported asthma and   - 1,815 mothers without self-reported asthma   163 mothers did not have genotype data;  78 did not pass sample-wise quality control  1,721 mothers with non-missing genotype, age, and asthma status   - 131 mothers with self-reported asthma and - 1,590 mothers without self-reported asthma   43 related individuals excluded  1,678 unrelated individuals with non-missing genotype, age, and asthma status   - 129 mothers with self-reported asthma and - 1,549 mothers without self-reported asthma |
| --- |

## Supplementary Table 1 Power calculations

| Minor Allele Frequency | Odds ratio | Power (under an additive model) | Minor Allele Frequency | Odds ratio | Power  (under an additive model) |
| --- | --- | --- | --- | --- | --- |
| (Significance threshold: 0.05) | | | (Significance threshold: 5x10^-8^) | | |
| 0.05 | 1.050 | 0.0532 | 0.05 | 1.050 | 0.0000 |
|  | 1.100 | 0.0625 |  | 1.100 | 0.0000 |
|  | 1.150 | 0.0778 |  | 1.150 | 0.0000 |
|  | 1.200 | 0.0990 |  | 1.200 | 0.0000 |
|  | 1.250 | 0.1258 |  | 1.250 | 0.0000 |
|  | 1.300 | 0.1581 |  | 1.300 | 0.0000 |
|  | 1.350 | 0.1956 |  | 1.350 | 0.0000 |
|  | 1.400 | 0.2377 |  | 1.400 | 0.0000 |
|  | 1.450 | 0.2838 |  | 1.450 | 0.0000 |
|  | 1.500 | 0.3331 |  | 1.500 | 0.0000 |
| 0.25 | 1.050 | 0.0625 | 0.25 | 1.050 | 0.0000 |
|  | 1.100 | 0.0991 |  | 1.100 | 0.0000 |
|  | 1.150 | 0.1590 |  | 1.150 | 0.0000 |
|  | 1.200 | 0.2401 |  | 1.200 | 0.0000 |
|  | 1.250 | 0.3378 |  | 1.250 | 0.0000 |
|  | 1.300 | 0.4448 |  | 1.300 | 0.0001 |
|  | 1.350 | 0.5530 |  | 1.350 | 0.0004 |
|  | 1.400 | 0.6545 |  | 1.400 | 0.0010 |
|  | 1.450 | 0.7436 |  | 1.450 | 0.0023 |
|  | 1.500 | 0.8171 |  | 1.500 | 0.0048 |
| 0.50 | 1.050 | 0.0664 | 0.50 | 1.050 | 0.0000 |
|  | 1.100 | 0.1138 |  | 1.100 | 0.0000 |
|  | 1.150 | 0.1898 |  | 1.150 | 0.0000 |
|  | 1.200 | 0.2894 |  | 1.200 | 0.0000 |
|  | 1.250 | 0.4039 |  | 1.250 | 0.0001 |
|  | 1.300 | 0.5222 |  | 1.300 | 0.0003 |
|  | 1.350 | 0.6339 |  | 1.350 | 0.0008 |
|  | 1.400 | 0.7315 |  | 1.400 | 0.0020 |
|  | 1.450 | 0.8110 |  | 1.450 | 0.0045 |
|  | 1.500 | 0.8720 |  | 1.500 | 0.0092 |

Power calculations were performed using QUANTO,^3^ specifying the available sample size (129 cases, 1,549 without asthma), allele frequencies between 0.05 and 0.5, and a range of effect sizes (odds ratios) consistent with those observed in the most recent multi-ancestry GWAS of asthma^4^, assuming an additive model.

## Supplementary Table 2 Proxies in the East Asian GBMI GWAS (at P-value<5x10^-6^)

| Proxy ID | Original SNP ID | Original SNP Effect Allele | Proxy correlated alleles | R2 | Distance (kb) |
| --- | --- | --- | --- | --- | --- |
| 2:8441735:C:G | 2:8442248:A:G | G | G=C, A=G | 0.995 | -513 |
| 2:24945954:A:G | 2:24943566:A:G | G | G=A, A=G | 1.000 | 2388 |
| 2:102931534:C:T | 2:102931612:C:T | T | T=C, C=T | 1.000 | -78 |
| 2:204584456:A:T | 2:204582623:C:T | T | T=A, C=T | 0.965 | 1833 |
| 2:242700770:A:G | 2:242698640:A:G | G | G=A, A=G | 1.000 | 2130 |
| 4:123132492:A:G | 4:123067808:A:G | G | G=A, A=G | 0.992 | 64684 |
| 5:110407507:C:T | 5:110401872:C:T | T | T=C, C=T | 0.500 | 0.4995 |
| 5:131995964:A:G | 5:131995843:C:T | T | T=A, C=G | 0.957 | 121 |
| 5:141494934:C:T | 5:141495139:C:T | T | T=C, C=T | 1.000 | -205 |
| 7:3146081:C:T | 7:3166865:A:G | G | G=C, A=T | 0.779 | -20784 |
| 7:20557179:A:G | 7:20560996:C:T | T | T=A, C=G | 0.983 | -3817 |
| 9:92226172:C:G | 9:92228559:G:T | T | T=C, G=G | 1.000 | -2387 |
| 10:9061370:C:T | 10:9052742:A:G | G | G=C, A=T | 0.993 | 8628 |
| 11:76292573:A:T | 11:76299649:A:C | C | C=A, A=T | 0.916 | -7076 |
| 15:61066516:C:T | 15:61068347:A:G | G | G=C, A=T | 0.764 | -1831 |
| 16:27354531:C:G | 16:27355362:A:G | G | G=C, A=G | 0.996 | -831 |
| 17:7786699:G:T | 17:7782130:C:T | T | T=G, C=T | 1.000 | 4569 |

proxy RSID coordinates and Original SNP ID – chromosome number:position:effect allele:other allele;

SNP – single nucleotide polymorphism

Correlated alleles – show which proxy allele is correlated with the original SNP effect allele

R2 – measure of the degree of linkage disequilibrium with the index SNP

## Supplementary Table 3 Weights and risk alleles for construction of the genetic risk score, derived from GBMI asthma GWAS^4^

| rsID | SNP ID | Risk allele | Weight | P-value |
| --- | --- | --- | --- | --- |
| rs1289273 | 1:226909233:A:G | G | 0.814 | 1.059x10^-07^ |
| rs13416555 | 2:8441735:C:G | C | 0.993 | 1.336x10^-11^ |
| rs17046490 | 2:24945954:A:G | A | 0.830 | 2.132x10^-06^ |
| rs72823632 | 2:102931534:C:T | C | 0.894 | 2.719x10^-06^ |
| rs4675360 | 2:204584456:A:T | A | 0.939 | 4.845x10^-13^ |
| rs35305862 | 2:242700770:A:G | A | 1.696 | 2.938x10^-17^ |
| rs35570272 | 3:33047662:G:T | T | 0.764 | 5.857x10^-09^ |
| rs4505848 | 4:123132492:A:G | G | 0.764 | 4.834x10^-09^ |
| rs2289276 | 5:110407507:C:T | T | 1.482 | 2.400x10^-28^ |
| rs20541 | 5:131995964:A:G | A | 0.864 | 4.568x10^-10^ |
| rs10068717 | 5:141494934:C:T | T | 0.665 | 8.142x10^-07^ |
| rs9270911 | 6:32572202:C:T | T | 1.134 | 2.977x10^-18^ |
| rs10266726 | 7:3146081:C:T | T | 0.737 | 1.969x10^-07^ |
| rs7780222 | 7:20557179:A:G | A | 0.804 | 1.671x10^-08^ |
| rs16902848 | 8:129399732:C:T | C | 0.705 | 1.300x10^-06^ |
| rs7040995 | 9:92226172:C:G | G | 0.694 | 5.670x10^-07^ |
| rs2589559 | 10:9061370:C:T | C | 1.754 | 6.512x10^-26^ |
| rs10995249 | 10:64396916:C:T | C | 0.749 | 9.834x10^-09^ |
| rs7126418 | 11:76292573:A:T | T | 0.758 | 2.125x10^-08^ |
| rs11639084 | 15:61066516:C:T | C | 1.382 | 1.685x10^-16^ |
| *rs17293632* | ***15:67442596:C:T*** | ***T*** | ***1.568*** | ***3.559x10^-06^*** |
| rs3024548 | 16:27354531:C:G | G | 1.141 | 8.924x10^-18^ |
| rs3744253 | 17:7786699:G:T | T | 0.937 | 1.165x10^-08^ |
| rs883770 | 17:38063381:C:T | C | 1.371 | 1.634x10^-20^ |
| rs2671654 | 17:47468011:A:G | A | 0.855 | 1.302x10^-07^ |
| rs57631119 | 18:45473449:C:T | T | 0.802 | 5.292x10^-07^ |
| rs10416530 | 19:9129660:C:T | C | 0.903 | 1.372x10^-09^ |

SNP ID – chromosome number:base-pair position:effect allele:other allele;

GRCh37 Genome Reference Consortium Human Build 37

Risk allele – the allele associated with an increased or decreased risk of a trait (i.e., asthma)

Weights are transformed beta values from the GBMI EAS asthma GWAS^4^ for each SNP. Betas represent the effect size (log odds ratio), indicating how much each copy of the effect allele contributes to the risk or development of the trait (in this case, asthma). For the genetic risk score (GRS), these betas were transformed so that a one unit increase in the GRS corresponded to one weighted allele, i.e., the betas were transformed so that for a given individual their minimum possible score for the GRS was 0 and the maximum was 54 (2 alleles multiplied by 27 variants, which was the number of SNPs studied). To transform the GRS such that a 1-unit increase corresponds to one weighted allele, each SNP's effect size (beta) is scaled by dividing it by the sum of all betas.

## Supplementary Table 4 Comparing estimates and direction of effects of the 27 variants tested in a candidate variant association analysis of self-reported asthma in mothers of the CLHNS cohort with the 37 top signals in GBMI EAS subgroup

| CHR:POS | EAS GWAS | | | CLHNS | | | Effect Direction |
| --- | --- | --- | --- | --- | --- | --- | --- |
|  | **EAF** | **OR [95% CI]** | **P-value** | **EAF** | **OR [95% CI]** | **P-value** |  |
| 1:203067614:T:TTG | 0.052 | 0.84 [0.80-0.89] | 1.222e-09 | - | - | - | + |
| 1:155708763:A:AAAATAAATAAATAAATAAAT | 0.631 | 1.14 [1.09-0.19] | 2.025e-08 | - | - | - | + |
| 2:242700770:A:G | 0.136 | 0.86 [0.83-0.89] | 2.629e-17 | - | - | - | + |
| 2:103051474:T:TA | 0.425 | 1.10 [1.07-1.12] | 1.08E-15 | - | - | - | + |
| 2:204570139:A:G | 0.476 | 0.92 [0.90-0.94] | 2.205E-13 | - | - | - | + |
| 2:8438693:C:T | 0.266 | 0.92 [0.89-0.94] | 5.863E-12 | - | - | - | + |
| 3:188133163:C:T | 0.595 | 0.92 [0.90-0.95] | 3.987E-11 | - | - | - | + |
| 3:121992825:C:T | 0.529 | 1.07 [1.05-1.10] | 2.094E-09 | - | - | - | + |
| 3:33047662:G:T | **0.494** | **1.07 [1.05-1.09]** | **5.857E-09** | **0.331** | **0.90 [0.69–1.20]** | **0.481** | **+,+** |
| 4:123333177:C:T | 0.497 | 1.07 [1.05-1.10] | 1.908E-10 | - | - | - | + |
| 5:110401872:C:T | **0.366** | **1.14 [1.11-1.16]** | **2.40E-28** | **0.243** | **1.07 [0.80–1.44]** | **0.630** | **+,-** |
| 5:131995964:A:G | 0.684 | 0.93 [0.91-0.95] | 3.705E-10 | - | - | - | + |
| 5:71822150:C:CA | 0.487 | 0.94 [0.92-0.96] | 4.05E-08 | - | - | - | + |
| 6:32432500:A:G | 0.111 | 1.33 [1.28-1.37] | 7.666E-58 | - | - | - | + |
| 6:31319391:A:C | 0.547 | 1.09 [1.07-1.12] | 3.059E-14 | - | - | - | + |
| 6:31831944:C:T | 0.247 | 1.10 [1.07-1.13] | 1.445E-13 | - | - | - | + |
| 6:29977869:C:T | 0.146 | 1.11 [1.08-1.14] | 2.285E-11 | - | - | - | + |
| 6:30809864:A:G | 0.373 | 1.08 [1.05-1.10] | 2.463E-10 | - | - | - | + |
| 6:32974268:C:T | 0.520 | 0.94 [0.92-0.96] | 1.134E-08 | - | - | - | + |
| 6:135399243:C:T | 0.216 | 1.08 [1.05-1.11] | 1.598E-08 | - | - | - | + |
| 7:20533171:C:T | 0.261 | 0.92 [0.90-0.94] | 3.23E-10 | - | - | - | + |
| 9:4980756:G:GT | 0.299 | 1.08 [1.05-1.11] | 5.377E-10 | - | - | - | + |
| 9:92202495:C:T | 0.286 | 1.08 [1.05-1.10] | 5.249E-09 | - | - | - | + |
| 9:4148717:C:G | 0.321 | 1.07 [1.05-1.10] | 8.651E-09 | - | - | - | + |
| 9:117490194:T:TC | 0.259 | 1.08 [1.05-1.10] | 2.674E-08 | - | - | - | + |
| 10:8940394:A:G | 0.596 | 0.88 [0.86-0.91] | 2.526E-28 | - | - | - | + |
| 10:64397538:C:T | 0.553 | 0.94 [0.92-0.96] | 7.004E-09 | - | - | - | + |
| 10:8115362:A:C | 0.506 | 0.94 [0.92-0.96] | 1.919E-08 | - | - | - | + |
| 11:76299649:A:C | **0.372** | **1.07 [1.04-1.09]** | **2.125E-08** | **0.311** | **1.03 [0.78–1.36]** | **0.847** | **+,-** |
| 12:56401085:A:G | 0.793 | 0.91 [0.88-0.93] | 1.074E-12 | - | - | - | + |
| 12:57497005:C:T | 0.220 | 1.09 [1.06-1.12] | 1.159E-09 | - | - | - | + |
| 14:69311341:A:G | 0.400 | 0.93 [0.91-0.95] | 1.149E-09 | - | - | - | + |
| 15:61068347:A:G | **0.171** | **0.89 [0.86-0.91]** | **1.685E-16** | **0.235** | **0.75 [0.55–1.04]** | **0.083** | **+,+** |
| 16:27358203:A:G | 0.664 | 1.11 [1.09-0.14] | 2.407E-18 | - | - | - | + |
| 17:38004929:G:GATTT | 0.265 | 0.88 [0.86-0.91] | 3.551E-21 | - | - | - | + |
| 17:7782130:C:T | **0.203** | **1.08 [1.05-1.12]** | **1.165E-08** | **0.110** | **0.74 [0.47–1.18]** | **0.207** | **+,+** |
| 19:9141159:A:C | 0.349 | 1.09 [1.07-1.12] | 1.168E-12 | - | - | - |  |

Chr – chromosome, Pos – base-pair position; GRCh37 – Genome Reference Consortium Human Build 37; OR– odds ratio; 95% CI – confidence interval;

The threshold for statistical significance was P=1.35x10^-3^, calculated as 0.05 divided by 37, the number of SNPs tested

Effect direction: Symbols denote direction of effect estimates from CLHNS and EAS GWAS presented in the table, in the same order. ORs are aligned at the same effect allele (‘+’ means consistent direction of effect, ‘-’ means opposite direction of effect)

References

1 Lange, L. A. *et al.* Genome-wide association study of homocysteine levels in Filipinos provides evidence for CPS1 in women and a stronger MTHFR effect in young adults. *Human Molecular Genetics* **19**, 2050-2058, doi:10.1093/hmg/ddq062 (2010).

2 Manichaikul, A. *et al.* Robust relationship inference in genome-wide association studies. *Bioinformatics* **26**, 2867-2873, doi:10.1093/bioinformatics/btq559 (2010).

3 Gauderman, W. J. Sample Size Requirements for Association Studies of Gene-Gene Interaction. *American Journal of Epidemiology* **155**, 478-484, doi:10.1093/aje/155.5.478 (2002).

4 Tsuo, K. *et al.* Multi-ancestry meta-analysis of asthma identifies novel associations and highlights the value of increased power and diversity. *Cell Genomics* **2**, 100212, doi:<https://doi.org/10.1016/j.xgen.2022.100212> (2022).
